# Properties of face localizer activations and their application in functional magnetic resonance imaging (fMRI) fingerprinting

**DOI:** 10.1371/journal.pone.0214997

**Published:** 2019-04-23

**Authors:** Lena Schwarz, Benjamin Kreifelts, Dirk Wildgruber, Michael Erb, Klaus Scheffler, Thomas Ethofer

**Affiliations:** 1 University Department of Psychiatry and Psychotherapy, University Hospital Tuebingen, Tuebingen, Germany; 2 Department for Biomedical Magnetic Resonance, University Hospital Tuebingen, Tuebingen, Germany; 3 Magnetic Resonance Centre, Max-Planck-Institute for Biological Cybernetics, Tuebingen, Germany; Virginia Polytechnic Institute and State University, UNITED STATES

## Abstract

Functional localizers are particularly prevalent in functional magnetic resonance imaging (fMRI) studies concerning face processing. In this study, we extend the knowledge on face localizers regarding four important aspects: First, activation differences in occipital and fusiform face areas (OFA/FFA) and amygdala are characterized by increased activation while precuneus and medial prefrontal cortex show decreased deactivation to faces versus control stimuli. The face-selective posterior superior temporal sulcus is a hybrid area exhibiting increased activation within its inferior and decreased deactivation within its superior part. Second, the employed control stimuli can impact on whether a region is classified in group analyses as face-selective or not. We specifically investigated this for recently described cytoarchitectonic subregions of the fusiform cortex (FG-2/FG-4). Averaged activity across voxels in FG-4 was stronger for faces than objects, houses, or landscapes. In FG-2, averaged activity was only significantly stronger in comparison with landscapes, but small peaks within this area were detected for comparison versus objects and houses. Third, reproducibility of individual peak activations is excellent for right FFA and quite good for right OFA, whereas within all other areas it was too low to provide valid information on time-invariant individual peaks. Finally, the fine-grained spatial activation patterns in right OFA and FFA are both time-invariant within each individual and sufficiently different between individuals to enable identification of individual participants with near-perfect precision (fMRI fingerprinting).

## Introduction

Faces constitute a privileged visual cue, enabling humans to recognize individuals from a virtually unlimited number of alternatives and extract a wealth of social (e.g. emotions, attitudes) and non-social (e.g. age, gender) information. The neural foundations of face perception consist of a distributed network of brain areas [1]. The ‘core areas for face perception’ are the fusiform face area (FFA, [2] and the occipital face area (OFA, [3]), which are thought to extract invariant aspects of faces, and the posterior superior temporal sulcus (pSTS, [4]), processing changeable aspects of faces. The ‘extended network for face perception’ [5] includes the amygdala subserving emotion processing [6] as well as the medial prefrontal cortex (mPFC), precuneus/posterior cingulate cortex (PC/pCC) and temporoparietal junction (TPJ) which are part of the default mode network (DMN, [7]), but process also information regarding familiarity [8] or mental states [9].

The neural system supporting these various aspects of face perception exhibits a strong interindividual variability which cannot be fully accounted for by structural normalization techniques (e.g. [10]). A frequently employed approach to tackle this problem is the application of face localizers that classify regions/voxels as ‘face-selective’ if they show stronger activation to faces than control stimuli (reviewed in [11]) during functional magnetic resonance imaging (fMRI). This method has been applied in numerous neuroimaging studies and thus the definition of selectivity in face localizers forms the foundation for a considerable amount of research on face perception.

Here, we examine face localizers in two groups of healthy subjects, a large group of 80 participants scanned once (“group 80x1”) and a smaller group of 5 participants each scanned 5 times on the same paradigm on 5 separate days (“group 5x5”) with regard to four important aspects: First, some face processing regions belonging to the DMN display deactivations during cognitive tasks. Thus, it is expected that these brain areas do not show task-positive effects (i.e. stronger activation versus baseline to faces than control stimuli) found in the sensory areas but rather task-negative effects (i.e. weaker deactivation versus baseline to faces than control stimuli). The distinction of these different processes is missed by block-designs without an explicit baseline condition as subtracting activation during perception of faces and control stimuli can mathematically yield identical results. This is particularly relevant for the pSTS which we aim to disambiguate from the adjacent presumably task-negative TPJ.

Second, it has been noted that the employed control stimuli can have a considerable impact on definition of face-selectivity [12]. Therefore, we systematically compare the results obtained by different sets of control stimuli. We focus particularly on the recently described subregions FFA-1 and FFA-2 [13] regarding their responses to different stimuli and their exact location with respect to cytoarchitectonic maps [14].

Third, an assumption in the use of fMRI localizers is a temporally stable spatial distribution of face-selectivity. Previous studies showed that the individual localization of the activation peak is fairly stable for the FFA and OFA [15, 16], but this has not been addressed for other face processing areas, such as pSTS/TPJ and amygdala.

Finally, we tested whether the spatial activation across voxels or regions is time-invariant and individually characteristic enough to enable identification of individuals. This methodology has been recently introduced as ‘functional connectome fingerprinting’ [17] paving the way for new insights into individual brain architecture: Identification accuracies close to the precision of fingerprints convincingly demonstrate that the activation profile is at the same time strongly variable across and robust within participants. Moreover, fMRI fingerprinting could help to disambiguate regions which are driven by external stimuli (and thus reveal stable activations enabling identification of single participants with high precision) from those which additionally depend on internal states (e.g. attention, mood, task) which might vary across scanning sessions.

## Methods

### Participants

In total, data of 85 healthy participants were analysed in this study. All participants were right-handed as determined by the Edinburgh Handedness Inventory[18] and had normal or corrected to normal vision. 80 healthy volunteers participated once in a face localizer fMRI experiment (37 females, mean age (standard deviation): 27.3 (7.9) years). This group is referred to as “group 80x1”. The other five healthy volunteers (3 females, mean age 25.6 years, ranging from 22 to 28 years) were scanned five times with the same face localizer paradigm on five separate days (intervals between scanning sessions ranging from 1 to 32 days, mean 7.5 days). This group is referred to as “group 5x5”. The study conformed to the code of Ethics of the World Medical Association (Declaration of Helsinki) and the study protocol was approved by the ethics committee of the University of Tuebingen. All participants gave written informed consent prior to participating.

### Stimulus material and experimental design

Selection of stimulus categories and experimental design were adapted from previous studies on face processing [2, 19] and contained four categories (see S1 Fig): The category human faces (f) contained 20 photographs of students (10 female, 10 male, front-view, neutral to friendly expression) displaying their faces and necks against a white background. The category houses (h) consisted of photographs of 20 two- or three-storied buildings displayed against a white background. The category objects (o) displayed photographs and drawings of 20 different objects (e.g., spoon, chair, hat, umbrella) against a white background. The category landscapes (l) showed photographs of 20 different outdoor locations (e.g., mountains, hills, coastlines, lakes). The stimuli were presented at 10.3° × 10.3° visual angle using a block-design with 20 stimuli per block (presentation duration per stimulus: 300 ms + 500 ms fixation cross, total duration of each block: 20 x 800 ms = 16 s). Within each block one repetition occurred randomly during the first and a second one within the second half of the block. To keep participants attentive to the stimuli, they were engaged in a one-back-matching-task (i.e., they were asked to press a button with their right hand whenever a stimulus was directly repeated). Each category occurred eight times in pseudorandomized order across the experiment with full randomization of the 20 stimuli within each block resulting in 9.5 minutes scanning time. In addition, the experimental design included a 27 s rest period during which a fixation cross was presented.

### Image acquisition

All participants were examined in a 3 Tesla TRIO Tim scanner (Siemens, Erlangen, Germany) using a 20 channel head coil. Functional imaging data comprised 336 volumes acquired with an echo-planar-imaging sequence covering the whole cerebral cortex (30 axial slices acquired in sequential descending order, slice thickness 4 mm + 1 mm gap, TR = 1.7 s, TE = 30 ms, voxel size = 3 x 3 x 4 mm, flip angle = 90°). For distortion correction, a fieldmap (TR = 400 ms, TEs = 5.19 and 7.65 ms, slice thickness = 3 mm, flip angle = 60°) was acquired prior to each functional run. A high-resolution T1-weighted anatomical 3D magnetization prepared rapid acquisition gradient echo (MPRAGE, FOV = 256 x 256 mm, 1 mm isotropic voxel size, TR = 2.2 s, TE = 2.92 ms, TI = 900 ms, flip angle = 9°) data set was obtained to enable precise normalization of the functional scans.

### Analysis of behavioural data

Behavioural data are reported as mean ± standard error of the mean. Responses to the target stimuli were classified as hits if they occurred 300–1500 ms after stimulus onset. Reaction times and hit rates of the 80x1 group were analysed using a one-way-ANOVA testing for within-participant effects of category (faces, houses, landscapes, objects). Corresponding behavioural data of the 5x5 group were analysed using a two-way-ANOVA testing for within-participant effects of category, of session number, and of their interaction. Non-sphericity was accounted for using the Greenhouse-Geisser correction [20].

### Analysis of fMRI data

The images were analysed using statistical parametric mapping software (SPM8, Wellcome Department of Imaging Neuroscience, London, UK). The first five functional volumes were discarded to allow for T1 equilibrium. Preprocessing included correction for differences in slice acquisition time with the middle slice as reference, realignment and unwarping on basis of the static field map to correct for movement as well as static and movement dependent field distortions [21], coregistration to the high-resolution anatomical image, normalization [10] to Montreal Neurological Institute (MNI) space (resampled voxel size: 3 x 3 x 3 mm), and spatial smoothing with an isotropic Gaussian filter of 8 mm full width at half maximum. Statistical analysis was based on the general linear model. Four different regressors were defined for the four stimulus categories, using a boxcar function corresponding to block onset and length convolved with the hemodynamic response function. Low frequency components were filtered out by applying a high-pass-filter with cut-off-frequency of 1/128 Hz. The error term was modelled as a first-order autoregressive process (AR coefficient = 0.2) plus white noise to account for serial autocorrelations within the data.

The contrasts f > h, f > o, and f > l as well as the main effect of faces (f versus baseline) were computed at the individual level. For the 80x1 group, the resulting contrast images and the main effect of faces were entered into a second-level random-effects analysis and thresholded using a voxel-wise family-wise error (FWE) corrected height threshold of p < 0.05. The contrast images were subjected to a conjunction analysis f > h ∩ f > o ∩ f > l based on the conjunction null hypothesis ([22]. The main effect of faces was employed to differentiate whether face-selective responses reflected increased activations or decreased deactivations to faces versus control stimuli.

The automated anatomical labelling (AAL, [23]) atlas was used to anatomically define regions of interest (ROIs) for the FFA within the posterior and middle part (posterior to y = -35) of the fusiform gyrus (FG), the OFA within the inferior occipital gyrus (IOG), the pSTS/TPJ consisting of the superior and middle temporal gyrus as well as angular gyrus, all posterior to Heschl’s gyrus (i.e. posterior to y = -29) to exclude predominantly auditory areas, and the amygdala. To examine the functional response profile of subregions of the fusiform gyrus, the cytoarchitectonic areas FG-2 and FG-4, [14], we employed the Anatomy toolbox [24], thresholding at a minimum cytoarchitectonic probability of 40%, to extract averaged responses (beta values) from these areas for group analysis. Furthermore, we also examined whether activation peaks within these cytoarchitectonic areas can be identified at the level of individual subjects (p < 0.001, uncorrected) and tested whether the amount of activated voxels significantly exceeds chance level (one-sided one-sample t-test).

### Within-subject reproducibility

Within the ROIs determined by AAL, we registered the number of peak activations in the 80x1 group based on conjunction analysis f > h ∩ f > o ∩ f > l. Peak activations within the ROIs were defined by voxels with a minimum T-value of 1.65 (p < 0.05, uncorrected) and a higher T-value than each of the surrounding 26 voxels (i.e., within a cube of 3 x 3 x 3 voxels). We employed two approaches in the 5x5 group, likewise using the conjunction analysis f > h ∩ f > o ∩ f > l, to assess the reproducibility of such peak activations: In the first approach, we employed the first imaging run to define activation peaks and investigated whether these peaks can be reliably found in the majority of subsequent imaging runs (i.e. whether an activation peak can be identified at the same or directly surrounding MNI coordinates in at least three of the four subsequent runs). In the second approach, we employed a MATLAB-implemented minimum variance agglomerative cluster algorithm (Ward’s clustering, for a review of this method see [25] to spatially group activation peaks across the five imaging sessions by using the MNI coordinates as features. We investigated whether activation peaks can be identified in at least four out of five runs within a cluster with an inner variance of 20 mm^2^ according to Ward’s agglomerative clustering which roughly corresponds to a sphere around the geometric mean of the grouped coordinates with a radius of 4–5 mm. Thus, while both approaches accept a comparable spatial variation of the peaks across runs, the clustering approach has the advantage that the definition of the center of the volume does not solely depend on activation in one index run. Finally, we also determined the percentage of reproducibly activated voxels across runs within the ROIs at the statistical thresholds of p < 0.001, p < 0.01, and p < 0.05 (uncorrected) by averaging first the results of all 10 pair-wise comparisons of the five sessions within each participant and then across participants Overlap was computed as R_ij_ = 2*V_ij_/(V_i_+V_j_) where R_ij_ is the proportion of overlapping voxels, V_ij_ the number voxels commonly activated in runs _i_ and _j_, V_i_ the number of voxels activated in run i and V_j_ the number of voxels activated in run j (analogously to [16]). The reported standard error of the mean refers to the variability across participants who exhibited at least one activated voxel in at least one of the five imaging runs. Subjects were excluded if there was no activated voxel within the ROI in any session.

### Classification using support vector machines

Support vector machines (SVMs) based on the software “libsvm” [26] (available at https://www.csie.ntu.edu.tw/~cjlin/libsvm) were used to test whether activations in the FFA, OFA, pSTS/TPJ, and amygdala of both hemispheres can be employed to identify single participants based on their individual characteristic response patterns of the contrasts f > h, f > l, f > o, separately or combined, as well as their conjunction. In the group 5x5, the SVMs were trained using voxel-by-voxel activation patterns within bilateral FFA, OFA, and pSTS/TPJ (based on fMRI data with and without smoothing) as features. Additionally, we trained SVMs using maximum t-values within each of these ROIs, all regions combined, coordinates of these maxima, or both t-values and coordinates of these maxima as features. To avoid disproportionate influence of single features (i.e. domination of attributes in greater numeric ranges), all feature values were standardized (i.e. subtraction of their mean and division by their standard deviation [27]. We employed a multiclass leave-one-out nested cross-validation procedure (i.e., the imaging run to be classified was not included in the training) based on freely available software (https://sites.google.com/site/kittipat/libsvm_matlab). The multiple classes (i.e. the five participants) were handled by pairwise comparison and the standard voting mechanism implemented in libsvm. Chance level was thus 20%. We computed the 95% confidence interval based on the binomial inverse cumulative distribution function implemented in Matlab ([28]. Accuracies were significantly above chance (p < 0.05) if they amounted to at least 32%.

### Classification with hyperplanes (fMRI fingerprinting)

In addition, we tested whether identification of participants can also be achieved based on a single imaging run per participant. To this end, we examined whether we can identify each of the 5 participants of the 5x5 group if we employ only one of their five imaging runs versus one imaging run of a participant of the 80x1 group as training data (5 * 5 * 80 = 2000 combinations). The data of the other 4 imaging runs of each participant of the 5x5 group to be identified are used as test data resulting in altogether 8000 classifications which were based on a separating hyperplane defined in point normal form with the normal vector connecting the coordinates given by the features of the two training data and the point being the geometrical centre between these coordinates. It was then examined whether the test data were situated on the same side of the hyperplane as the feature coordinates of this participant’s training data. Chance level was thus 50% and accuracies were significantly above chance (p < 0.05) if they reached 68% or more.

## Results

### Behavioural data

The participants of the 1x80 group successfully responded to 88.3 ± 1.1% (faces: 90.0 ± 1.2%, houses: 87.8 ± 1.4%, landscapes: 88.1 ± 1.5%, objects: 87.2 ± 1.4%) of the one-back trials within the predefined time interval (300–1500 ms) with a mean reaction time of 543.4 ± 7.6 ms. The univariate ANOVAs in the 80x1 group did not reveal a significant effect of category (faces, objects, houses, and landscapes) using either reaction times (F(3,224) = 1.70, p = 0.17) or hit rates (F(2,183) = 2.66, p = 0.07) as dependent variable. In the 5x5 group, the 4x5 ANOVA also revealed no significant effect of category (F(1,5) = 0.48, p = 0.55), session number (F(2,8) = 1.89, p = 0.21), or interaction between session number and category (F(2,8) = 0.78, p = 0.49) on reaction times either. The corresponding analysis with hit rates did also not reveal any effect of category (F(1,4) = 1.87, p = 0.24), session number (F(2,7) = 0.78, p = 0.48), or interaction between session number and category (F(2,7) = 0.82, p = 0.46). These results indicate comparable difficulty of the employed one-back matching task across categories without relevant learning effects across imaging sessions which represents an important prerequisite for comparability of stimulus types and stability of brain activity in different imaging runs.

### Group fMRI activation

**Fig 1:**
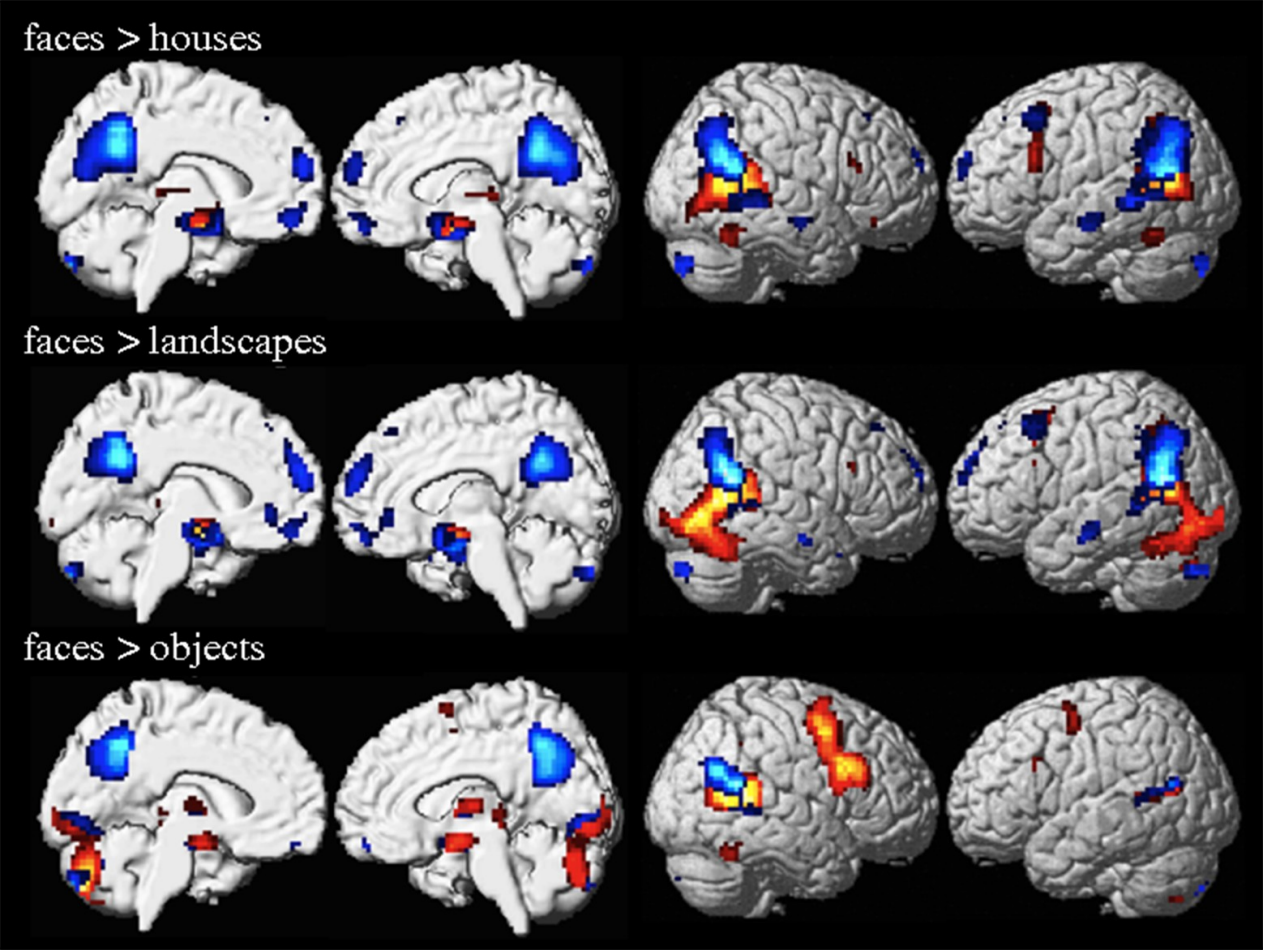
Group 80x1 analysis activation maps for the different contrasts. Activation maps for the contrasts a) f > h, b) f > l, and c) f > o, thresholded at p<0.05 FWE at voxel level, are rendered on the lateral and medial surface of the SPM standard brain template. Areas which are driven by enhanced activation and decreased deactivation to faces are shown in red/yellow and blue/light blue, respectively.

**Fig 2:**
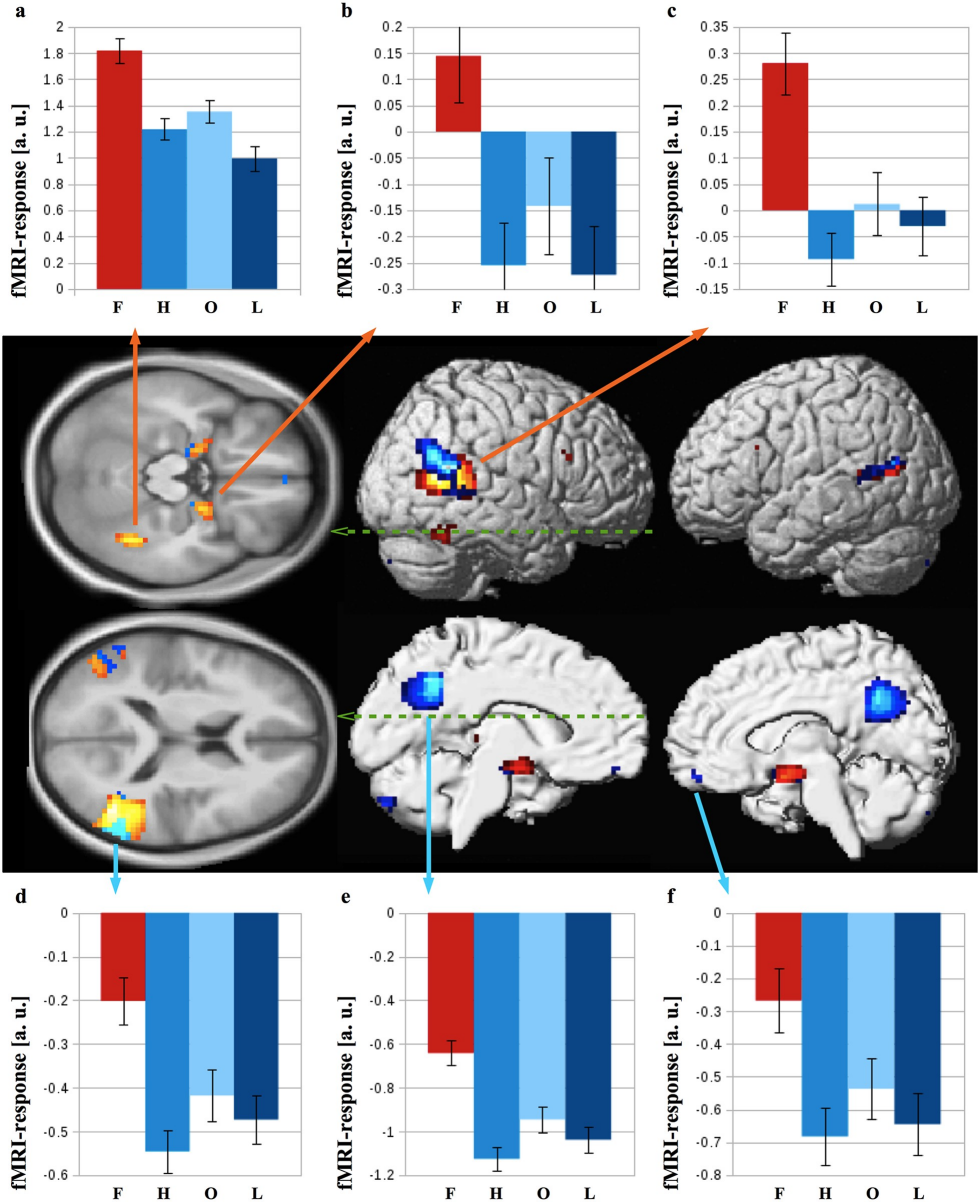
Group 80x1 analysis conjunction and per subject averaged beta estimates per condition and region. The results of the conjunction analysis f > h ∩ f > l ∩ f > o, thresholded at p<0.05 FWE at voxel level, are shown on lateral and medial surface of the SPM standard brain template as well as transversal slices (z = -18, top, and z = 12, bottom). Brain areas which are driven by enhanced activation and decreased deactivation to faces are shown in red/yellow and blue/light blue, respectively. Extracted beta estimates (mean ± standard error of the mean, a.u. = arbitrary units) from right fusiform cortex (a), right amygdala (b), and the inferior part of the right pSTS/TPJ (c) which resulted from enhanced activation are shown in the upper panels. Correspondingly, responses from the superior part of the pSTS/TPJ (d), PC/pCC (e) and MPFC (f) which resulted from decreased deactivation are presented in the lower panels.

**Fig 3:**
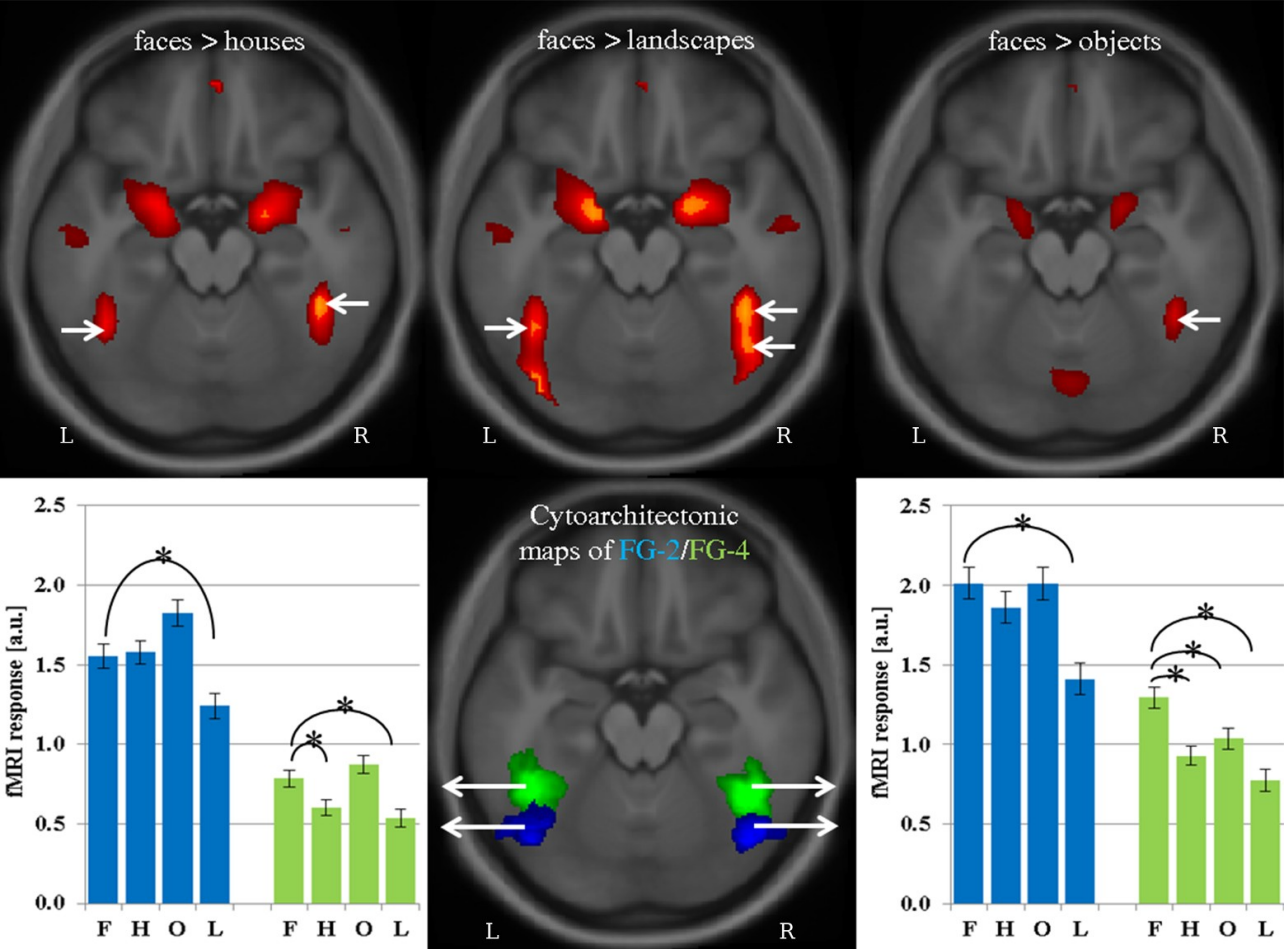
FFA-1 and FFA-2 in different contrasts and single conditions in group 80x1. Activation maps for f > h, f > l, f > o projected onto a transversal slice (z = -18) of the averaged normalized brain of the study participants (upper panels). Activation peaks are marked with white arrows. Cytoarchitectonic maps for FG-2 (blue areas) and FG-4 (green areas) are shown in the lower central panel. Extracted beta estimates (mean ± standard error of the mean, a.u. = arbitrary units) obtained from left and right FG-2 (blue bars) as well as FG-4 (green bars) are shown in the left and right lower panels, respectively. Significantly (p<0.001) smaller responses to houses, landscapes, and objects as compared to the face condition are denoted by asterisks.

Activation maps of the contrasts f > h, f > o, and f > l for the 80x1 group are shown in Fig 1a–1c. While the three contrasts yielded similar activations, some important differences should be noted. Contrasting faces versus houses or landscapes resulted in bilateral FFA activations, but only the right FFA reacted stronger to faces than objects. In f > l, activation extends posterior in the IOG and merges with activation in pSTS. Brain areas identified by the conjunction analysis (f > h ∩ f > o ∩ f > l, see Methods) within the 80x1 group are presented in Table 1 and Fig 2. In the right FG as well as bilateral amygdala and the inferior part of the pSTS/TPJ area this effect was driven by significantly stronger activation to faces than other stimuli (red-yellow clusters and panels a-c in Fig 2). In bilateral precuneus and medial prefrontal cortex as well as the superior part of the pSTS/TPJ the contrast in the conjunction analysis resulted from significantly weaker deactivation to faces than other stimuli (see blue-light blue clusters and panels d-f in Fig 2). Activation characteristics of cytoarchitectonically (see Methods) defined regions-of-interest (ROIs) within the fusiform gyrus are shown in Fig 3. In the right fusiform cortex, the contrast f > l revealed an anterior peak in FG-4 (x = 42, y = -45, z = -21) and a posterior peak in FG-2 (x = 42, y = -60, z = -18). Only one activation peak in right FG-4 was observed for f > h (x = 42, y = -45, z = -21) and f > o (x = 42, y = -48, z = -21). In the left fusiform cortex, one activation peak within FG-4 was observed for f > h and f > o (both peaks at x = -42, y = -54, z = -21). Evaluation of averaged activity across voxels revealed significantly stronger responses to faces as compared to all three control conditions (all p < 0.001, all T(79) > 6.7) for the right FG-4. The left FG-4 elicited significantly stronger responses to faces than houses or landscapes (both p < 0.001, T(79) > 5.5), but not objects. Averaging of responses across voxels in right and left FG-2 resulting in significantly stronger to faces than landscapes (both p < 0.001, T(79) > 6.7), but not the other two control conditions. Evaluation of individual, activation maps (thresholded at p < 0.001, uncorrected), however, showed significantly (p < 0.05) more activated voxels as compared to chance level for FG-2 and FG-4 of both hemispheres for all three contrasts (see Table 2).

**Table 1:** Areas showing significantly stronger activation resp. significantly weaker deactivation to faces than to all other conditions (conjunction analysis f>h and f>o and f>l) in group 80x1.

*Increased activation*	MNI-coordinates	Z score	cluster size*
Right middle temporal gyrus / right superior temporal gyrus / right angular gyrus	45–57 18	> 8.00	412
Right fusiform gyrus / inferior temporal gyrus	42–48–21	> 8.00	51
Right amygdala / right hippocampus	21–3–15	7.58	68
Left amygdala / right hippocampus	-18–6–15	6.68	47
Left middle temporal gyrus	-51–69 15	5.68	25
Left middle temporal gyrus / left superior temporal gyrus	-60–51 12	4.95	18
*Decreased deactivation*			
Right angular gyrus / right middle temporal gyrus / right superior temporal gyrus	45–60 24	> 8.00	217
Right precuneus / left precuneus / left posterior cingulum	3–60 39	> 8.00	336
Left middle temporal gyrus	-51–69 18	5.53	24
Right gyrus rectus / left gyrus rectus	6 54–15	5.41	12
Left cerebellum	-18–81–39	5.12	24
Left middle temporal gyrus / left superior temporal gyrus	-60–54 12	5.05	14

*p < 0.05, corrected at voxel level

**Table 2:** Average size (mean ± standard error of the mean) of individual activation peaks in mm^3^ across subjects in right and left FG2 and FG4 for each of the three control conditions.

	faces > houses	faces > objects	faces > landscapes
Right FG 2	48.6 ± 10.8 mm^3^	37.8 ± 11.6 mm^3^	52.9 ± 13.5 mm^3^
Left FG 2	48.3 ± 9.7 mm^3^	44.8 ± 9.2 mm^3^	62.6 ± 13.5 mm^3^
Right FG 4	34.6 ± 8.9 mm^3^	38.1 ± 10.3 mm^3^	59.4 ± 14.6 mm^3^
Left FG 4	35.1 ± 9.2 mm^3^	28.6 ± 8.1 mm^3^	48.3 ± 13.2 mm^3^

### Individual activation peaks

The number of activation peaks identified within the anatomically predefined ROIs in each individual participant of the 80x1 group is given in Table 3, revealing a considerable variation across participants and regions. While most participants exhibited at least one activation peak within both middle-to-posterior FGs (thus an “FFA”) and the right OFA, no activation peak was identified for the left OFA or the amygdala in the majority of participants. The right pSTS/TPJ typically exhibited at least four activation peaks. Interestingly, more than half of the participants (52.5%) revealed at least one peak that was driven by stronger activation and one peak which resulted from weaker deactivation to faces than other stimuli. In the left pSTS/TPJ, the number of peaks strongly varied across participants, accompanied by an overall lower activation strength in this area as compared to its right hemispheric homologue.

**Table 3:** Number of peaks across subjects in the 80x1 group.

Region	% participants having the respective number of peaks:				
	0	1	2	3	4/+
Right FFA	20.00	53.75	22.50	3.75	0.00
Left FFA	23.75	52.50	22.50	1.25	0.00
Right OFA	43.75	42.50	12.50	1.25	0.00
Left OFA	68.75	28.75	2.50	0.00	0.00
Right pSTS	1.25	10.00	12.50	15.00	61.25
Left pSTS	11.25	16.25	27.50	16.25	28.75
Right Amygdala	72.50	27.50	0.00	0.00	0.00
Left Amygdala	76.25	22.50	1.25	0.00	0.00

### Within-participant reproducibility of individual activation peaks

**Fig 4:**
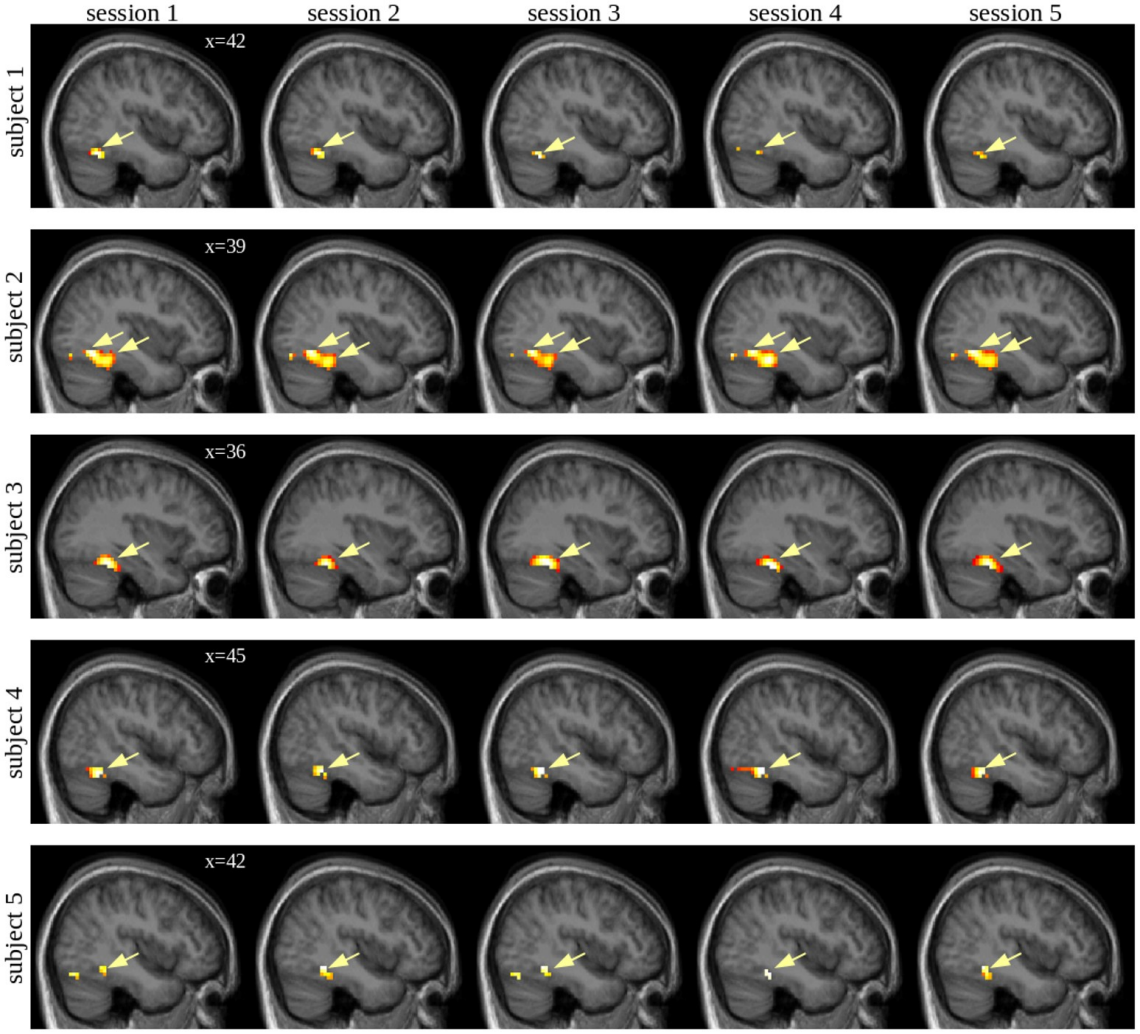
Right FFA peaks per subject across session in group 5x5. In each row, the individual conjunctions f >h ∩ f >o ∩ f >s of sessions 1 to 5, masked by the right FG ROI are shown in an exemplary sagittal slice. The consistent peaks are marked by yellow arrows.

To assess whether individual activation peaks are reproducible across imaging sessions, we examined whether peaks obtained in the first imaging run can also be identified in subsequent imaging runs of the 5x5 group, as given in Table 4. Individual activation maps of the right FFA are shown in Fig 4. In two participants, stable activation peaks were found directly at the border between the ROIs of the posterior FG and anterior IOG. In these cases, the peaks were assigned to the OFA because the peak maximum was situated in this area in the majority of imaging runs. The highest reproducibility was identified for the right FFA. For this region, the maximum peak of the first run was always consistently found across the subsequent imaging sessions and the number of peaks obtained in the first run corresponded to the results of the independent cluster analysis across runs. In the right OFA, the reproducibility was acceptable and agglomerative cluster analysis always identified one reproducible peak across runs. However, this peak was missed in one participant during the first run. The reproducibility was much lower in the left FFA as only about half of the peaks found in the first run were successfully identified in subsequent runs or the cluster analysis. In line with the results of the 80x1 group, four peaks were identified on average in the right pSTS/TPJ during the first imaging run, and cluster analysis identified on average 3.2 stable peaks across runs. However, taking the first run as standard, on average only one of its peaks could be reproduced and this peak was not necessarily the peak showing the highest t-score in the first run. In the left pSTS, the left OFA, and both amygdala individual peaks were only identified in a minority of participants and/or the identified peaks were not reproducible. The results regarding reproducibility of activated voxels within the ROIs yielded similar results as the evaluation of activation peaks: For all evaluated statistical thresholds, the highest number of reproducibly activated voxels was found for the right FFA followed by the right OFA (see Table 5). The percentage of reproducibly activated voxels found in our study for right FFA und right pSTS was very similar as compared to Engell and McCarthy (2013) who also examined these regions [16].

**Table 4:** Percentage of overlapping voxels within the ROI across sessions in group 5x5. Values are given in % +/- standard error of the mean and number of included subjects in brackets.

Region / Threshold	P<0.001 (uncorrected)	P<0.01 (uncorrected)	P<0.05 (uncorrected)
right FFA	47.9 +/- 14.6 (5)	58.0 +/- 11.2 (5)	68.8 +/- 7.3 (5)
left FFA	25.4 +/- 9.9 (4)	37.7 +/- 9.1 (4)	38.9 +/- 11.5 (5)
right OFA	34.9 +/- 17.0 (5)	46.5 +/- 15.6 (5)	58.6 +/- 11.4 (5)
left OFA	34.1 +/- 0.0 (1)	29.1 +/- 19.6 (2)	42.9 +/- 19.1 (2)
right pSTS	20.5 +/- 8.5 (5)	29.5 +/- 8.15 (5)	32.8 +/- 7.4 (5)
left pSTS	0.1 +/- 0.1 (3)	4.2 +/- 4.0 (4)	8.0 +/- 4.1 (5)
right Amygdala	0.0 +/- 0.0 (1)	2.4 +/- 2.4 (4)	8.1 +/- 1.8 (4)
left Amygdala	0.0 +/- 0.0 (0)	0.0 +/- 0.0 (3)	0.9 +/- 0.2 (3)

**Table 5:** Peak reproducibility within the 5x5 group.

Region	Reproducibility of peak maximum	Number of peaks in first run: mean (range)	Number of reproducible peaks: mean (range)	Number of peaks obtained by cluster analysis: mean (range)
Right FFA	5 / 5	1.2 (1–2)	1.2 (1–2)	1.2 (1–2)
Left FFA	3 / 4	1.4 (0–2)	0.6 (0–2)	0.8 (0–1)
Right OFA	3 / 4	1.0 (0–2)	0.6 (0–1)	1.2 (1–2)
Left OFA	1 / 1	0.2 (0–1)	0.2 (0–1)	0.2 (0–1)
Right pSTS	1 / 5	4.0 (2–6)	1.0 (0–3)	3.2 (2–4)
Left pSTS	0 / 2	0.8 (0–3)	0.0 (0–0)	0.2 (0–1)
Right Amygdala	0 / 2	0.4 (0–1)	0.0 (0–0)	0.4 (0–1)
Left Amygdala	0 / 1	0.2 (0–1)	0.0 (0–0)	0.0 (0–0)

### Pattern classification

Employing SVMs we determined whether the fine-grained activity within the predefined ROIs of the face perception network or activation across these regions can be employed for identification of fMRI participants within the 5x5 group (see Table 6). Voxel-wise Multivariate Pattern Analysis (MVPA) of the bilateral FFA and OFA enabled 100% classification accuracy (cut-off for significance: accuracy ≥ 32%), with the exception of the bilateral OFA in smoothed f > h where it was slightly lower (95%). Classification was less accurate, but still in a remarkable range, in the pSTS (64–96%). The amygdalae were rather poorly suited for classification (accuracy 16–44%). Higher accuracies were obtained in all areas (except for the amygdalae) if no smoothing was applied to the images. Furthermore, we tested whether successful identification of individuals is also possible by restricting the available information across regions to the maximum t-score, coordinates of these maxima, or their combination as features (see Table 6, last three rows). This approach yielded accuracies of 64–100%. More information (i.e., using several contrasts and/or both peak t-scores and coordinates) improved the identification accuracy.

**Table 6:** SVM classification accuracy in %, with classification based on smoothed (unsmoothed) data within the 5x5 group.

MVPA	conjunction	faces > houses	faces > objects	faces > landscapes	Combination
Right fusiform	100 (100)	100 (100)	100 (100)	100 (100)	100 (100)
Left fusiform	100 (100)	100 (100)	100 (100)	100 (100)	100 (100)
Right inferior occipital	100 (100)	96 (100)	100 (100)	100 (100)	100 (100)
Left inferior occipital	100 (100)	96 (100)	100 (100)	100 (100)	100 (100)
Right pSTS	96 (96)	92 (92)	80 (92)	92 (96)	92 (92)
Left pSTS	84 (96)	84 (84)	64 (76)	72 (84)	92 (96)
Right Amygdala	28 (40)	32 (24)	24 (16)	24 (44)	44 (40)
Left Amygdala	36 (28)	24 (16)	28 (20)	32 (20)	32 (16)
Regions combined					
Peak T scores	80	76	84	64	88
Peak coordinates	84	100	96	84	100
Peak T scores and coordinates	92	96	100	92	100

### Classification with hyperplanes

Finally, we examined whether successful identification of individuals can be achieved if only one imaging run per participant is available as training data. To this end, we tested whether we can identify each of the 5 participants of the 5x5 group if we employ only one of their five imaging runs versus one imaging run of a participant of the 80x1 group as training data). The data of the other 4 imaging runs of each participant of the 5x5 group to be identified served as test data and we calculated separating hyperplanes to determine the classification accuracy using analogous features as employed in the SVM approach (cut-off for significance: accuracy ≥ 68%, see Table 7). Even though slightly less accurate than training SVMs over several sessions, qualitatively very similar results with remarkably high accuracy were achieved for voxel wise MVPA in FFA and OFA (91–99%), medium accuracy for the pSTS (64–88%), and performance at chance level for the amygdalae (42–53%). Again, accuracy improved when using unsmoothed data in all areas except for the amygdalae. Classification with information expressed across brain areas (peak t-scores and/or coordinates) yielded 76–91% correct classifications.

**Table 7:** Simple hyperplane classification accuracy in %, with classification based on smoothed (unsmoothed) data.

MVPA	conjunction	faces > houses	faces > objects	faces > landscapes	combination
Right fusiform	92 (99)	91 (98)	93 (98)	93 (99)	95 (99)
Left fusiform	90 (99)	91 (98)	85 (98)	91 (98)	92 (99)
Right inferior occipital	92 (97)	90 (97)	92 (96)	94 (99)	95 (98)
Left inferior occipital	90 (96)	90 (94)	89 (96)	95 (96)	96 (98)
Right pSTS	73 (79)	74 (88)	67 (72)	70 (77)	73 (79)
Left pSTS	73 (77)	68 (73)	64 (66)	64 (67)	70 (71)
Right Amygdala	52 (48)	48 (44)	47 (46)	51 (45)	49 (45)
Left Amygdala	49 (48)	42 (44)	53 (49)	51 (45)	47 (45)
Regions combined					
Peak T scores	83	76	81	78	80
Peak coordinates	81	81	89	82	86
Peak T scores and coordinates	87	83	91	86	89

## Discussion

The current neuroimaging study allowed us to address several key questions of functional localizers. Specifically, we aimed to determine differential contributions of increased activation versus decreased deactivation within the face processing network. In addition, we explored to which extent definition of face-selectivity is dependent on the employed control condition. Finally, we investigated the stability of individual activation peaks in predefined ROIs of key areas for face perception and whether the activation patterns within these areas are characteristic and robust enough to identify individual participants with high precision.

Behavioral data indicated similar reaction times and hit rates without significant differences for the four different types of visual stimuli and across imaging runs indicating comparable task difficulty without relevant learning effects due to repeated participation in the same paradigm. These behavioral findings represent important prerequisites for comparability of stimulus types and stability of brain activity in different imaging runs.

Contrasting face-related brain activity to different control conditions yielded the well-known areas of the core and extended network for face perception. However, closer inspection revealed important differences regarding which regions are classified as face-selective. This dependency on the control condition and stability of face-related activity is discussed separately for each of the examined regions of the face processing network before we address the potential of multivariate techniques for the identification of individuals due to their characteristic pattern within this network.

### Fusiform face area

While contrasting faces versus houses or landscapes yielded bilateral FFA activations, only the right FFA reacted stronger to faces than objects which is concordant with previous results [12]. Peak activations in the right FFA were highly reproducible corroborating previous studies [15, 16] while left hemispheric peaks were reproducible only in roughly half of the cases. In line with previous studies [2, 19], we used different objects (e.g. spoon, hat, iron, etc..) rather than different versions of one class of objects. While the composition of different objects in this category allows a better generalization of the resulting contrast, strong differences in shape, semantic associations, and possible applications occurring within in each block of presented objects, but not in blocks with faces might influence brain activation. This is a possible explanation for the observation that the left FFA is missed in the contrast faces versus objects, but not in the contrast faces versus houses. Future studies should directly address this question and determine whether the left FFA is activated in a conjunction analysis with different versions of stimuli across several object classes (e.g. faces > spoons ∩ faces > hats ∩ faces > irons …), but not in the contrast faces versus mixed objects. The contrast faces versus landscapes revealed two activation peaks in the right posterior and mid fusiform cortex replicating reports on two face-selective clusters within the fusiform gyrus which have been named FFA-1 and FFA-2 [13, 16, 29]. Overlay of the two peaks identified in our study with cytoarchitectonic maps confirmed that FFA-1 and FFA-2 are situated in FG-2 and FG-4, respectively [14] replicating recent results [30]. The posterior peak (FFA-1 in FG-2) was only observable in the faces > landscapes contrast if activity was averaged across all voxels in FG-2. Analysis of individual activation maps, however, revealed significantly more activated voxels than expected at chance level for all three contrasts. These findings indicate that face-selectivity of FFA-1 in FG-2 is restricted to a subregion of this cytoarchitectonically defined area and thus missed in group analyses averaging across voxels of this region.

### Occipital face area

The definition of face-selective voxels within the IOG is strongly dependent on the control condition with highly significant effects for contrasts versus landscapes but occurring to a much lesser extent for the other two contrasts. A strikingly similar observation was made by McCarthy and Engell (2013) who reported activation maps for the contrasts faces versus scenes and houses in a large group of participants (see Fig. 1 in [16]). The contrast faces versus landscapes revealed an activation peak in the lateral IOG which accords with previous reports on OFA activations [31]. It is difficult, however, to disambiguate the OFA in this contrast from the neighbouring object-selective lateral occipital cortex (LOC, [32]. A similar problem has been pointed out by Rossion and colleagues (2012) who discouraged using scrambled faces as sole control condition as this classifies the LOC as “face-selective” [12]. Contrasts versus landscapes are also burdened with this problem as the resulting cluster partially extends into voxels which react strongest to objects (o>f ∩ o>h ∩ o>l, data of this contrast not shown). A possible explanation for this spill-over into the LOC for faces > landscapes is that faces have clear edges and are additionally displayed against a uniform background making them more “object-like” than landscapes which do not have natural borders.

### Posterior superior temporal sulcus/temporoparietal junction

Perception of faces resulted in enhanced activity within the pSTS/TPJ for all three control conditions replicating previous observations on face-selectivity posterior to the bifurcation of the STS [33, 34]. It has been proposed that activity in this region reflects decoding of changeable features including emotions [35–37], trustworthiness [38–40], or eye gaze [41–43]. The pSTS and the adjacent TPJ have also been associated with moral judgments (e.g.[44–46]) and theory-of-mind (e.g. [47–49]). In our study, the pSTS/TPJ cluster was composed of a ventral part that was driven by stronger activations and a dorsal part driven by weaker deactivations to faces than other stimuli. Therefore, a conjunction approach (i.e. faces > control stimuli ∩ faces > fixation cross) has been previously employed [50] to include only voxels where activation differences between faces and control stimuli are driven by stronger activations. As decreased deactivations to faces as compared to the employed control stimuli occurred consistently across participants as evidenced by the group analysis as well as the analysis of individual peaks (the majority of fMRI participants of the 80x1 group revealed at least one peak driven by decreased deactivation in right pSTS/TPJ), we think, however, that such approaches might exclude relevant brain activity related to face-processing. A possible way to better characterize specific functions of different peaks within the pSTS/TPJ could be to combine face localizer experiments with other imaging paradigms. A specific hypothesis to address in future studies would be whether peaks due to smaller deactivation correspond exactly to those elicited by moral judgment or theory-of-mind tasks [47–49].

### Amygdala

Both amygdalae showed activation to faces, but deactivation during all three employed control conditions confirming their long proposed role within the “extended network” for face processing [1] which has been particularly emphasized for evaluation of social information (for a review, see [51]). The contrasts faces versus control stimuli yielded activation clusters which extended slightly into the hippocampus and other neighbouring temporal lobe structures. Positive responses to faces were restricted to the amygdala while deactivations to control stimuli were additionally observed in neighbouring structures explaining why amygdala activations to faces were surrounded by a zone where activation differences are driven by smaller deactivation to faces than control stimuli. Although the activation results at group level were highly significant, the activations at individual level were not strong enough to define clear peaks within the amygdala for individual participants.

### Other brain areas

In addition, contrasting faces to each of the three classes of control stimuli revealed face-selective areas in the mPFC and PC/pCC which is in line with data from previous fMRI experiments relying on face localizers (e.g.[16, 34, 52]). These areas belong to the DMN [7] which is characterized by decreases of brain activity during cognitive task demands. In our study, we also observed decreased responses in these areas during the one-back task as compared to presentation of a fixation cross which were less pronounced for faces than control stimuli. It is unlikely that these deactivation differences are due to different levels of task demands as the four experimental conditions were characterized by similar hit rates and reaction times, but could signify automatic engagement in processes regarding face familiarity [8, 53].

Interestingly, a cluster within the right lateral superior prefrontal cortex was found if faces were contrasted with objects, but not if landscapes or houses were employed as baseline. Stronger activations to faces versus objects in this area have been observed before (e.g. [50]). So far, the functional significance of this finding is unclear. However, the fact that activation differences were restricted to one control condition questions that this area carries out face-specific processes.

### fMRI fingerprinting within the face areas

We demonstrated identification of participants with near-perfect accuracy using pattern analysis of activation in face-selective regions. This is even possible if classification is based on only one imaging run per participant (hyperplane classification). The best identification rates were obtained for the FFA and OFA (98–99%). We used the term fMRI fingerprinting for this method as it is characterized by a high between-subject discrimination (i.e. the subjects of the 5x5 group could be successfully identified versus the large library of face localizer scans of the 80x1 group) as well as a high within-subject reproducibility (i.e., this result is not dependent on which of the five imaging runs of each of the participants of the 5x5 group are included in the test data). A possible explanation for the lower accuracy found for pSTS/TPJ (71–79%) could be that face processing in this area depends on a self-referential context which is not necessarily stable across scanning sessions (i.e., the participant’s thoughts and attitudes towards the viewed faces) within areas of the DMN (e.g. the adjacent cortex around the TPJ). Within the amygdala, the identification accuracy was always around guess rate indicating that the spatial activation pattern is not characteristic for single participants.

Within the FFA, OFA, and pSTS/TPJ, higher accuracy rates were obtained for unsmoothed data indicating that smoothing results in a loss of fine-grained information rather than suppression of noise. Accuracy rates were similar for the three contrasts faces versus houses, objects, and landscapes indicating that classification was not merely driven by subtraction of a specific control condition. Interestingly, identification with comparably simple features obtained across regions (i.e. maximum t-score or coordinates of peak activations) also yielded accuracies which were considerably higher than guess rate providing a proof-of-principle that also relative activation differences across regions are specific for individual participants. However, this sparse information was not sufficient to enable identification with near-perfect accuracy as found for spatial activation patterns in the FFA or OFA.

### Limitations

The current study relied on standard methods for fMRI acquisition and data analysis. The motivation of this approach was to facilitate comparison with the vast majority of fMRI experiments typically using similar acquisition parameters and analysis tools. However, possible limitations of the current results regarding image resolution, application of spatial smoothing, and normalization procedures realigning individual brains standard templates for group analysis have to be acknowledged. Regarding image resolution previous methodological studies on the application of face localizers demonstrated that localization of activation peaks are relatively stable regardless of whether image acquisition occurs with relatively small (1.5–2 mm) or large voxels (3–4 mm) [54, 55]. It has been shown that application of spatial smoothing can result in merging of activation clusters in FFA-1 and FFA-2 in individual subjects [54, 55]. At group level, however, this merged activation cluster had still two separable peaks in our study and very similar results were obtained using smoothed or unsmoothed data indicating that application of spatial smoothing cannot explain why the more posterior FFA-1 exhibited face-selectivity only when contrasted versus landscapes, but not versus houses or objects. Previous methodological studies have shown that curvature-driven cortex based alignment (CBA) methods can result in better inter-individual correspondence of anatomical structures than nonlinear volumetric alignment (NVA) as applied in our study. It is important to notice, however, that this better inter-individual correspondence of structural anatomy results in higher overlap of functional activation profiles only for some areas, such as the frontal eye fields, while functional differences remain in other areas, such as the FFAs [56]. A recent study [57] investigated this issue for the FFA in more detail showing that CBA is superior to NVA in decreasing spatial variability across subjects for some cytoarchitectonic areas of the visual ventral stream, such as FG-4 where FFA-2 is located, but not for area FG-2 where FFA-1 is located. Therefore, we feel that the lack of face specificity in FFA-1 observed for the contrasts versus houses or objects at group level in our study cannot be explained by the applied standard NVA tool (3D normalization in SPM, [10]).

The reproducibility measures regarding localization of individual activation peaks are based on a small sample of participants. We found an excellent reliability for the right FFA and to a somewhat lesser degree for the right OFA which corroborates previous observations [15, 16, 58, 59]. The main inference, however, made on the basis of these reproducibility data is that localization of individual stable peaks fails for other face-selective areas, such as the pSTS/TPJ and amygdala, limiting their usefulness for these structures. To make this point, a small group is sufficient as a reliable face localizer should provide reproducible results in almost all participants which is clearly not the case. Moreover, any reproducibility findings have to be interpreted within the context of the employed experimental design as well as MRI scanner and sequences. We employed a 10-min block-design localizer with static stimuli at 3T which represents the most frequently applied procedure. Potential approaches to increase statistical power (and consequently possibly the reproducibility of individual peak responses) include application of dynamic stimuli [60, 61], higher field strength [62], fMRI at short TRs [63], or longer scanning time [64].

### Conclusions

Our results have important implications for applications of face localizer scans in future neuroimaging studies. First, we demonstrated that face-selectivity can be due to stronger activations or smaller deactivations to faces versus control stimuli and differentiation of these qualitatively different processes necessitates a baseline condition. This is especially important for the pSTS/TPJ which constitutes a hybrid area with its inferior part driven by increased activation and its superior part by decreased deactivation. Second, we demonstrated that the control condition has a strong influence on which regions are classified as ‘face-selective’. Importantly, FFA-1 in FG-2 was missed by group analysis some contrasts, while individual activation peaks could be identified indicating that face-selectivity is a property of subregions of FG-2, but not FG-2 as a whole. We argue against the use of landscapes as control stimuli to identify the OFA as this approach overestimates the face-selective effect within the IOG and results in spill-over of activations into the object-selective LOC. Third, the reproducibility of individual peak activations is excellent for the right FFA and quite good for the right OFA. The pSTS/TPJ, however, contains several peaks exhibiting similar t-scores limiting the usefulness of face localizers for this area. Finally, multivariate analyses revealed that the activation patterns within the FFA and OFA are characteristic and stable enough to allow near-perfect identification of participants underlining the stability of individual visual representations in sensory cortices.

## Supporting information

S1 Fig Design matrix of the fMRI face localizer experiment. Regressors from left to right are houses, faces, scenes, and objects blocks.
(TIF)

S1 File Blank copy of consent form. Original participant consent form (German).
(DOC)

S2 File Blank copy of consent form—English translation. Translated participant consent form (English).
(DOC)
