# Supplementary figures and images for: Properties of face localizer activations and their application in functional magnetic resonance imaging (fMRI) fingerprinting
Source: PLoS One. 2019 Apr 23;14(4):e0214997. doi: 10.1371/journal.pone.0214997 (PMC6478291; doi:10.1371/journal.pone.0214997)

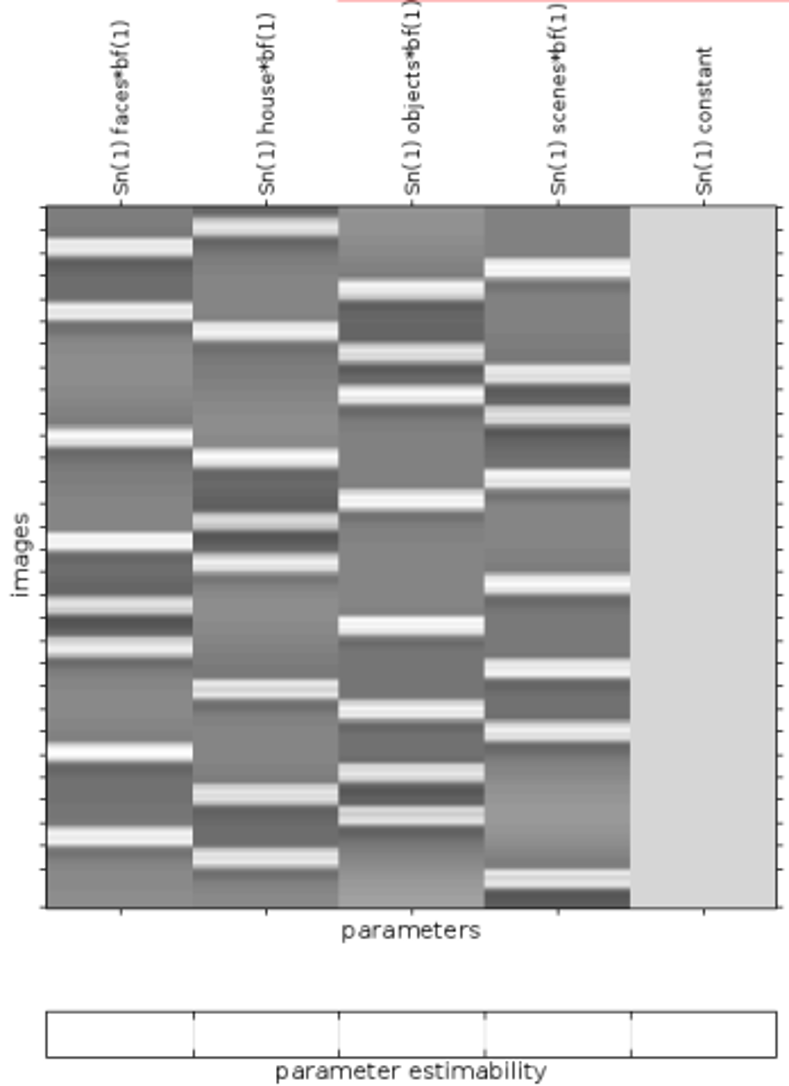

Supplement: S1 Fig — Regressors from left to right are houses, faces, scenes, and objects blocks. (TIF) [file pone.0214997.s001.tif]
