# Supplementary material for: Properties of face localizer activations and their application in functional magnetic resonance imaging (fMRI) fingerprinting
Source: PLoS One. 2019 Apr 23;14(4):e0214997. doi: 10.1371/journal.pone.0214997 (PMC6478291; doi:10.1371/journal.pone.0214997)
Supplement: S1 File — Original participant consent form (German). (DOC) [file pone.0214997.s002.doc]

| 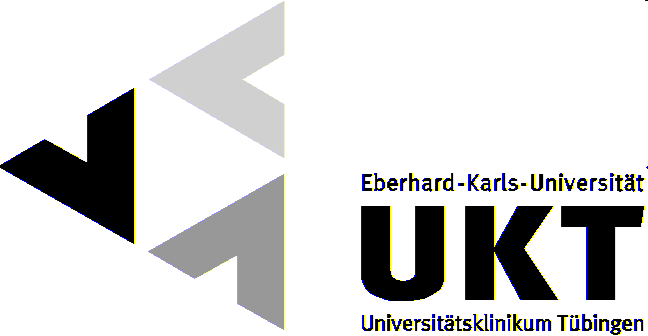 |  | **Einwilligungserklärung und Information zum Datenschutz** |
| --- | --- | --- |
|  |  | *Abteilung für biomedizinische Magnetresonanz, Otfried-Müller-Straße 51, 72076 Tübingen, Prof. Dr. Thomas Ethofer, Tel. 07071/29-87384* |

# Information zum Datenschutz

- Ihre im Rahmen der wissenschaftlichen Untersuchung erhobenen Daten werden vertraulich behandelt und ausschließlich in verschlüsselter Form weitergegeben. Die für die wissenschaftliche Untersuchung wichtigen Daten werden in verschlüsselter (pseudonymisiert, ohne Namensnennung) Form in einen gesonderten Dokumentationsbogen eingetragen.
- Die Zuordnung der verschlüsselten Daten zu Ihrer Person ist nur anhand einer Patientenliste möglich, die in einem ver­schlos­senen Schrank, getrennt von den Studienunterlagen aufbewahrt wird und nur dem Stu­dien­leiter und dem Ärztlichen Direktor der Abteilung zugänglich ist. Die Daten werden 10 Jahre nach Studienende vernichtet.
- Sollten Sie von der Studie zurücktreten, können Sie entscheiden, ob die bereits vorliegenden Daten vernichtet werden müssen oder weiterverwendet werden dürfen.
- Bei der Auswertung und Veröffentlichung werden ausschließlich verschlüsselte Daten verwendet.
- Alle an der Studie beteiligten Personen unterliegen der Schweigepflicht.

# Einwilligungserklärung

**Ich erkläre mich einverstanden**

- dass die im Rahmen der oben genannten Studie erhobenen Daten in der oben beschriebenen Weise gesammelt, ausgewertet und veröffentlicht werden.
- mit der freiwilligen Teilnahme an der oben genannten Studie.

**Ich wurde darüber informiert**

- dass ich jederzeit meine Daten beim Studienleiter einsehen kann.
- dass bei meinem Rücktritt aus der Studie die bis dahin erhobenen Daten vernichtet werden.
- dass ich mein Einverständnis jederzeit formlos und ohne Nachteile für meine Person widerrufen kann.

Alle Fragen zur Ziel, Dauer, Ablauf und Nutzen der Studie wurden zu meiner Zufriedenheit beantwortet.

|  |  |  |  |  |
| --- | --- | --- | --- | --- |
| Ort & Datum |  | Unterschrift |  | Name in Blockschrift (Proband) |

|  |  |  |  |  |
| --- | --- | --- | --- | --- |
| Ort & Datum |  | Unterschrift |  | Name in Blockschrift (Versuchsleiter) |
