# Supplementary material for: Properties of face localizer activations and their application in functional magnetic resonance imaging (fMRI) fingerprinting
Source: PLoS One. 2019 Apr 23;14(4):e0214997. doi: 10.1371/journal.pone.0214997 (PMC6478291; doi:10.1371/journal.pone.0214997)
Supplement: S2 File — Translated participant consent form (English). (DOC) [file pone.0214997.s003.doc]

| 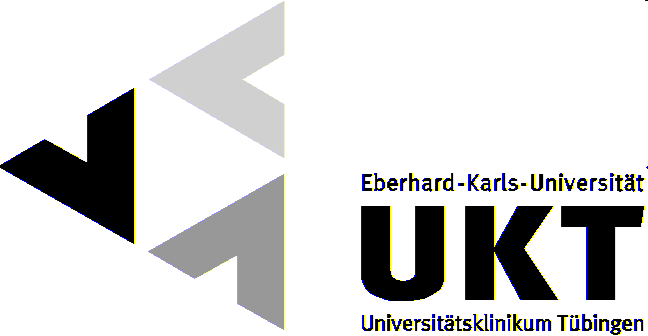 |  | **Consent form and information regarding data protection** |
| --- | --- | --- |
|  |  | *Department for Biomedical Magnetic Resonance, Otfried-Müller-Straße 51, 72076 Tübingen, Prof. Dr. Thomas Ethofer, Tel. 07071/29-87384* |

# Information concerning data protection

- Your data obtained during the scientific examination are being handled privileged and shared encrypted only. The data important for the scientific examination are being entered in a separate documentation sheet in encrypted (pseudonymized, without naming names) form.
- The allocation of the encrypted data to you is only possible with the aid of a participant list, which is stored in a locked cupboard separately from the study documents and which is accessible only to the principal investigator and the medical director of the department. Data will be deleted 10 years after the end of the study.
- Should you rescind the study, you can decide whether the already present data must be wiped out or can still be used.
- For analysis and publication only encrypted data are used.
- All staff involved in the study are subject to confidentiality obligation.

# Declaration of consent

**I declare my agreement**

- that the data obtained during the study are being collected, analyzed and published in the above described fashion.
- to voluntary participation in the study.

**I was informed**

- that I can inspect my data with the principal investigator at any time.
- that my data obtained to this time will be wiped out in case of my rescission from the study.
- that I can rescind my consent informally and without any detriment to myself at any time.

Alle Fragen zur Ziel, Dauer, Ablauf und Nutzen der Studie wurden zu meiner Zufriedenheit beantwortet.

All questions regarding aim, duration, procedure and benefit of the study were answered to my satisfaction.

|  |  |  |  |  |
| --- | --- | --- | --- | --- |
| Place & Date |  | Signature |  | Name in block letters (Proband) |

|  |  |  |  |  |
| --- | --- | --- | --- | --- |
| Place & Date |  | Signature |  | Name in block letters (Principal Investigator) |
